# The impact of teacher punishment intensity on parental trust in rural China: an experimental examination of a moderated mediation model

**DOI:** 10.3389/fpsyg.2025.1599092

**Published:** 2025-05-27

**Authors:** Chunhui Qi, Juan Guo, Yanfeng Liu, Zhen Zhang, Guoxiang Zhao

**Affiliations:** ^1^Faculty of Education, Henan Normal University, Xinxiang, China; ^2^Faculty of Education, Henan University, Kaifeng, China

**Keywords:** punishment intensity, violation severity, parental trust, trustworthiness, rural China

## Abstract

Pedagogical punishment refers to the educational behavior carried out by teachers to manage, instruct or correct students who violate rules, so as to promote the healthy development of students. In home-school partnerships, parents determine their level of trust in educators based on pedagogical punishment. Preliminary empirical evidence indicates that the punishment intensity impacts parents’ interpersonal trust, though the causal pathways remain to be elucidated. Utilizing a situational experimental design with 462 rural Chinese guardians (234 females, mean age is 40.86 years), this research establishes a causal relationship between pedagogical punishment intensity and parental trust. The analysis further evaluates trustworthiness perception as a mediator and student violation severity as a moderating variable. The findings demonstrate that teacher punishment not only directly strengthens parental trust but also indirectly increases it through perceived trustworthiness. Notably, the severity of student misconduct moderates the relationship between punishment intensity and parental trust, indicating that rigorous punishment behaviors in response to severe violations foster heightened perceived trustworthiness relative to less stringent interventions. This study offers critical insights to education professionals on the rationale and strategies for implementing appropriate punishment practices, thereby enhancing parental trust in educators.

## 1 Introduction

Home-school collaboration significantly enhances school teaching quality and fosters parental engagement in education, emerging as a vital element in international education reform (Hummel et al., 2023). Parental trust in teachers, a prerequisite for effective collaboration, is defined as parents’ willingness to delegate educational responsibilities to teachers based on perceived competence, benevolence, and integrity (Hiatt et al., 2023; Mayer et al., 1995). As the foundation of home-school partnerships, this trust supports the development of constructive family-school dynamics and promotes children’s holistic growth (Bormann et al., 2021; Niedlich et al., 2021; Shayo et al., 2021). Research across education, psychology, and sociology over the past 30 years has extensively explored strategies for cultivating reciprocal trust between families and schools (Rautamies et al., 2021; Santiago et al., 2016; Uitto et al., 2021). Studies employing mixed-method approaches reveal that most parents acknowledge teachers’ professionalism and demonstrate trust (Huang, 2022; Janssen et al., 2012; Schuster et al., 2025), though this trust varies significantly depending on individual, familial, institutional, and societal contexts (Adams and Christenson, 2000; Bower et al., 2011; Forsyth et al., 2006; Kikas et al., 2011, 2016; Lerkkanen and Pakarinen, 2021). Among these influencing factors, the role of teachers stands out as the most direct factor in building parents’ trust and has been a key focus for scholars.

When parents communicate with teachers, they frequently evaluate the trustworthiness of educators based on observable social cues such as teacher gender, management style, professional competence, and caring behavior (Kikas et al., 2011, 2016; Schuster et al., 2025). These factors influence parents’ decisions regarding the extent and nature of their trust in teachers. Among these observable indicators, teacher disciplinary actions are a particularly salient concern for families, schools, and society at large. Signaling theory suggests that individuals’ words and actions act as social signals, sharing actor’s moral character and reputation with observers (Connelly et al., 2011; Gintis et al., 2001). The implementation and manner of punishment also significantly impact bystanders’ trust judgments and choices concerning punishers. In the field of behavioral economics, numerous studies using economic game tasks have confirmed that third-party punishment can convey the moral reputation of the punisher, thereby influencing bystanders’ evaluations of trustworthiness and subsequent trust decisions (Jordan et al., 2016; Salcedo and Jimenez-Leal, 2024; Sun et al., 2023). Similarly, within organizational management contexts, episodic paradigm-based situational experiments have found that appropriate punishment can enhance perceptions of leadership credibility among employees or students, thereby increasing bystander trust (Wang and Murnighan, 2017). Conversely, inappropriate punishment from teachers can undermine students’ observer trust by diminishing perceived trustworthiness of teachers (Zhang and Qi, 2024). However, existing research has not adequately addressed the spillover effect of school discipline on parental trust, particularly within the context of basic education in rural areas. In 2023, the number of students enrolled in rural basic education in China reached 22.4 million, representing a significantly disadvantaged group that warrants immediate attention. On one hand, rural basic education is plagued by the inequitable distribution of educational resources, subpar educational quality, and a pronounced issue of excessive teacher discipline (Guo and Li, 2024). On the other hand, rural parents are constrained by limited economic development, often bearing substantial financial burdens (Ma et al., 2018). Additionally, their relatively low educational attainment hinders effective home-school collaboration (Xie and Postiglione, 2016). Thus, there is an urgent need to establish a robust home-school partnership to address these challenges.

**Figure 1:**
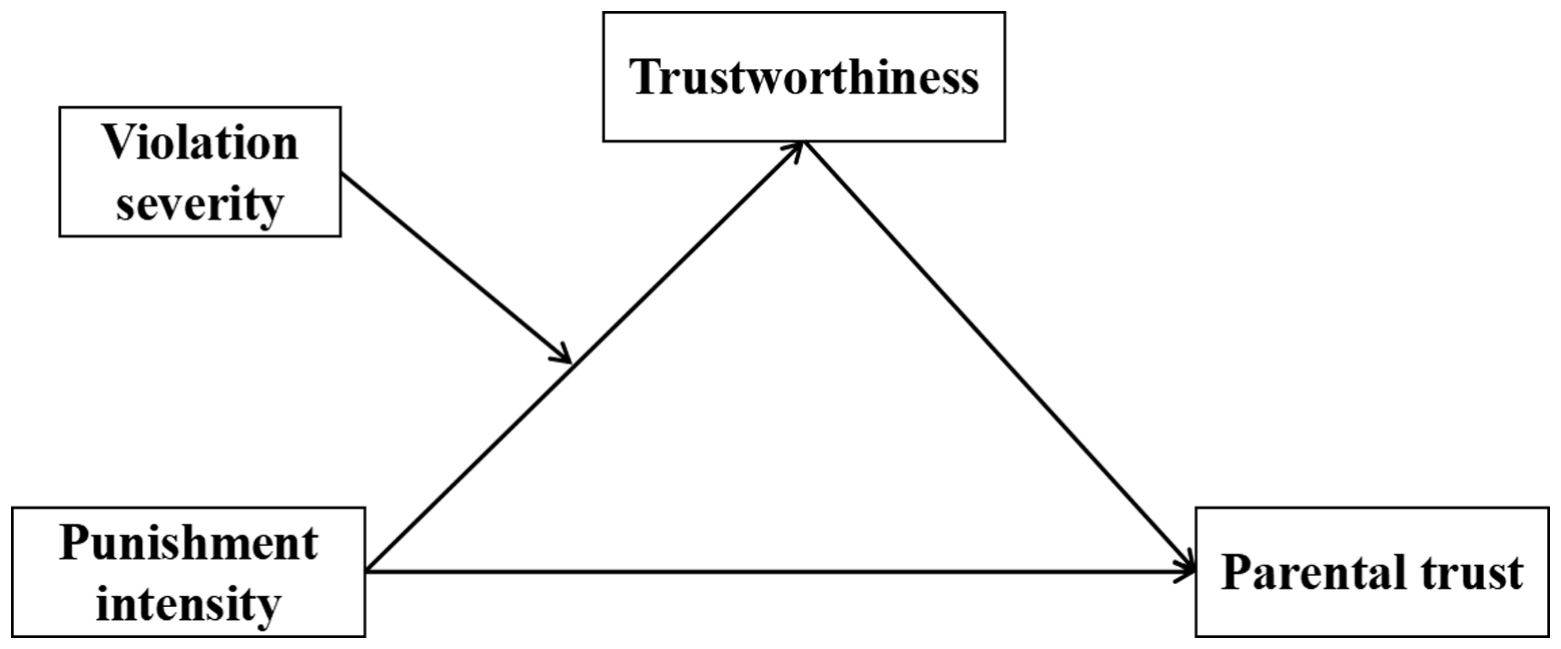
Research model.

Given that parental trust is essential for effective home-school cooperation, it is imperative to investigate how teacher disciplinary intensity influence parents’ trust in school management. This study developed a moderated mediation model to systematically investigate the impact of teacher disciplinary intensity, student violation severity, and trustworthiness on parental trust in rural China. The conceptual framework of this research model is illustrated in Figure 1. In this model, the punishment intensity refers to the severity of the disciplinary action. In China, it is categorized into three levels: mild punishment (e.g., verbal reprimand), relatively severe punishment (e.g., contacting parents), and extremely severe punishment (e.g., suspension or expulsion from school). Given that extremely severe disciplinary actions are relatively infrequent and cannot be solely enforced by teachers, the present study focuses exclusively on the first two types of disciplinary actions.

## 2 Literature review and research hypotheses

### 2.1 Punishment intensity and parental trust

In the context of school education, punishment serves as one of the most frequently employed intervention strategies by teachers to manage students’ problematic behaviors (Zhang and Qi, 2024). The gravity of imposed sanctions correlates with offense severity, extending from verbal admonishments to administrative actions including temporary suspensions and permanent expulsions. Such disciplinary interventions not only influence offenders’ cognitive processes and behavioral patterns but also shape the perceptions of observing students or parents concerning institutional authority and educational effectiveness (Sun et al., 2023; Wang and Murnighan, 2017; Zhang and Qi, 2024; Zhang et al., 2025a). Signal theory holds that individuals convey signals to others in various ways during social interactions to express their thoughts and values. Similarly, observers could also infer the personality qualities and reputation information of the transmitter based on the respective signals expressed by the transmitter. When the cost required for the signal is higher, the signal is also accurate and reliable (Connelly et al., 2011; Gintis et al., 2001). As a tool for fostering students’ compliant behaviors, punishment should focus on correcting and preventing problematic behaviors rather than assuming that greater intensity always yields better outcomes (Raihani and Bshary, 2019). Empirical investigations in organizational behavior demonstrate a curvilinear correlation between disciplinary intensity and stakeholder trust, wherein moderate interventions optimize trust consolidation while excessive leniency or harshness proves counterproductive (Wang and Murnighan, 2017; Xiao et al., 2019; Zhang and Qi, 2024). Based on these findings (Xiao et al., 2019; Zhang and Qi, 2024), we propose Hypothesis 1: There exists an inverted U-shaped relationship between the punishment intensity and parental trust.

### 2.2 Trustworthiness as a mediator

Trustworthiness represents an individual’s propensity to fulfill the positive expectations of others and serves as a critical antecedent variable for interpersonal trust (Zhang et al., 2024). Structurally, trustworthiness encompasses three dimensions: ability, benevolence, and integrity (Mayer et al., 1995). Extensive research has demonstrated that these three dimensions are robust predictors of interpersonal trust in various roles such as leaders, teachers, or administrators (Colquitt et al., 2007; Hiatt et al., 2023; Zhang and Qi, 2024). Additionally, Jordan et al. (2016) have shown through multiple studies that third-party punishment can serve as an effective indicator for conveying trustworthiness and reputation information about disciplinarians, thereby assisting bystanders in forming attitudes and impressions. Recent studies have further revealed that the signaling effect of trustworthiness in punishment transmission varies with changes in punishment intensity (Salcedo and Jimenez-Leal, 2024; Wang and Murnighan, 2017; Zhang and Qi, 2024). Specifically, within a certain range, bystanders’ perceived trustworthiness increases with higher disciplinary intensity; however, once this intensity exceeds a threshold, excessive discipline significantly diminishes bystanders’ trustworthiness judgments of the disciplinarian. Importantly, trustworthiness has been found to mediate the relationship between disciplinary intensity and bystander trust (Wang and Murnighan, 2017; Zhang and Qi, 2024). Based on these findings (Wang and Murnighan, 2017; Zhang and Qi, 2024), we propose Hypothesis 2: Compared with no punishment, both mild punishment and severe punishment can enhance perceived trustworthiness of teacher, thereby increasing parental trust. On the contrary, compared with mild punishment, severe punishment may reduce parents’ trust by weakening the perceived trustworthiness of teacher.

### 2.3 Violation severity as a moderator

Violation severity pertains to the assessment of the gravity of violations evaluated by analyzing the intention, frequency, and consequences of the deviant act (Eriksson et al., 2017; Peterson, 2024). When imposing disciplinary actions, it is imperative to holistically assess these dimensions to ensure alignment with the principle of proportionality in punishment—a cornerstone of administrative law that mandates penalties correspond to the gravity of the offense. This principle, widely endorsed by the public, resonates with the just deserts theory (JDT), which posits that sanctions must equitably reflect the transgression (Mooijman and Graham, 2018). Appropriately calibrated responses to minor infractions reinforce observers’ perceptions of the disciplinarian’s trustworthiness, whereas disproportionate or absent penalties may erode trustworthiness and provoke skepticism. Empirical studies corroborate that proportional sanctions enhance perceived legitimacy, while excessive leniency or severity diminishes it (Peterson, 2024; Wang and Murnighan, 2017; Zhang and Qi, 2024). For example, Zhang and Qi (2024) found that teachers’ appropriate responses to classroom discipline violations positively influence students’ trustworthiness in their judgment, while inappropriate responses can undermine this trustworthiness. Based on these findings (Wang and Murnighan, 2017; Zhang and Qi, 2024), we posit Hypothesis 3: The severity of student violations positively moderates the association between teachers’ disciplinary intensity and perceived trustworthiness. Specifically, when compared to no punishment, both mild and severe discipline can increase the perceived trustworthiness of teacher, specially for high violation conditions.

## 3 Method

### 3.1 Experimental design and participant

The current study employed a two-factor between-subjects experimental design with 3 levels of punishment intensity (no punishment, mild punishment, severe punishment) and 2 levels of violation severity (low-severity violation, high-severity violation), resulting in six distinct hypothetical scenarios. In this hypothetical experiment, a homeroom teacher observes that a student in her class has committed a classroom violation (either arriving 10 min late or skipping an entire class). The homeroom teacher must decide whether to impose disciplinary action on the offending student. If disciplinary measures are chosen, the options available to the teacher include verbal reprimand or contacting the parents for a meeting at the school. Based on pre-tests and prior research (Wang and Murnighan, 2017; Zhang and Qi, 2024), classroom violations were categorized into low-severity (being 10 min late) and high-severity (skipping a class). Punishment intensity was classified as no punishment, mild punishment (verbal reprimand), and severe punishment (contacting parents).

Utilizing an *a priori* power analysis, the necessary sample size was calculated using G*Power 3.1 software (Faul et al., 2009). For determining the sample size, we opted for the F-test within the ANOVA framework. With an assumed medium effect size of *f* = 0.25 and a significance level set at α = 0.05, it was determined that a minimum of 252 participants would be needed to achieve a statistical power of 1 − β = 0.95. This requirement corresponds to a minimum of 42 participants for each experimental condition. China’s basic education system is comparable to that of the United States, as both countries adopt the K-12 educational framework. The research was carried out in a junior middle school situated in Yichuan County, Luoyang City, Henan Province, China. From this school, six classes from the 8 grade were randomly chosen, and the parents of students in these classes were selected as the study participants. All the tests were done at the classroom level and parents are blind to study conditions. Each class’s parents were allocated to complete only one specific experimental condition. For this research, two matching disciplinary situation questionnaires were allocated to each classroom cohort, with instructions for students to return completed parental responses during the subsequent Monday’s school session. Following data validation procedures which excluded incomplete or unreliable entries from the initial 509 distributed surveys, 462 qualified responses were retained. All experimental conditions maintained a threshold of at least 59 validated returns. Adult respondents spanned ages 32–67 (*M* = 40.86 ± 5.18 years), with demographic composition showing 50.6% female representation, 47.6% agricultural hukou, and 3.0% transient households. The adolescent cohort, aged 12–16 (*M* = 13.39 ± 0.52 years), consisted of 50.0% female students and 4.3% non-only children.

A certified research associate administers questionnaires to all participating classrooms and gives detailed guidance. Participants receive assurance that all collected data remains anonymous, maintains strict confidentiality, and serves purely academic objectives. Students were instructed to take the research questionnaires home over the weekend and ensure that parents completed them as specified. The anticipated time allocation for responding to all survey items averages 15 min. Ethical clearance for this investigation was obtained from the Ethics Committee of the Faculty of Education, Henan Normal University, with full alignment to the Helsinki Declaration’s research ethics framework. Detailed specifications of the study’s operational variables accompanied by explanatory notes appear systematically organized in Table 1.

**Table 1:** The explanatory and descriptive statistics of variables in data analysis.

Type	Variable name	items	Variable description	M	SD
Predictor variable	Punishment intensity	The intensity of the teacher’s disciplinary approach	1 = no punishment; 2 = mild punishment; 3 = severe punishment	2.08	0.78
Moderator variable	Violation severity	The seriousness of students breaching school policies	1 = low-severity violation; 2 = high-severity violation	1.50	0.50
Mediation variable	Trustworthiness	Wang has always been a steadfast support for the child.	1 = Strongly disagree; 2 = more disapproval; 3 = basically agree; 4 = more agree; 5 = Strongly agree	4.18	0.93
		Wang is highly reliable.			
Outcome variable	Parental trust	I intend to delegate an important matter to Wang.		3.73	1.08
		I will assign the custody of my personal belongings to Wang.			
		I would like to share something about my child with Wang			
Control variable	Parental characteristics	Parental identity	1 = Biological father; 2 = Biological mother	1.51	0.50
		Parental age	Age at the time of the survey	40.86	5.18
	Student’s characteristics	Student’s gender	0 = Female; 1 = Male	1.50	0.50
		Student’s age	Age at the time of the survey	13.39	0.52
		Only-child status	1 = Only child; 2 = Not an only child	1.96	0.20
	Family characteristics	Hukou classification	1 = agricultural hukou; 2 = non-agricultural hukou	1.52	0.50
		Family mobility	1 = Non-mobile; 2 = Cross-township mobile	0.03	0.17

### 3.2 Measures

This investigation adopted a printed questionnaire distributed to parents of students in six separate distinct classes, with participants randomly allocated one of six experimental narratives to review and respond to. These narratives contained four core sections: demographic details of both the parents and their children, a hypothetical situation involving discipline, an evaluation of operational variables, and a parental assessment of disciplinary educators. By adopting this method, the study aimed to gather comprehensive data on parental perspectives regarding disciplinary practices. The demographic information provided context for the participants, while the hypothetical scenarios allowed for standardized responses. The operational assessment helped measure specific variables, and the social evaluation offered insights into parents’ views on teachers’ trustworthiness and interpersonal trust.

#### 3.2.1 Basic demographic variables of parent and their child

To account for individual variations within parent–child dyads, participants were requested to submit demographic data encompassing their gender, age, hukou classification, and family mobility, in addition to the child’s gender, age, and only-child status.

#### 3.2.2 Hypothetical scenario of teacher discipline

Drawing from the study by Zhang and Qi (2024), a hypothetical situation involving classroom discipline was presented to participants. In this scenario, it was communicated to the participants that their children had faced disciplinary action imposed by Teacher Wang, who serves as the head teacher, due to breaches of class regulations. Specifically, the children were either tardy by 10 min or missed an entire class session, which brought them to Teacher Wang’s attention. As the individual responsible for managing the class and in line with the school’s guidelines, Teacher Wang opted for one of three responses: notifying the parents (considered a severe punishment), issuing verbal warnings (a mild punishment), or choosing not to take any action (no penalty).

#### 3.2.3 Manipulation checks

We employed four operational test questions to evaluate participants’ perceptions of the severity of a child’s misconduct in disciplinary scenarios, as well as the appropriateness and intensity of the teacher’s disciplinary actions. The first two questions assessed participants’ views on the seriousness of the child’s violation, for example, “The child’s behavior contravenes school rules and regulations” and “The child’s behavior warrants disciplinary action”; the internal consistency coefficient for these items was 0.75. The third question evaluated participants’ perceptions of the severity of the discipline imposed, such as “The child has faced stringent disciplinary actions.” The fourth question gauged participants’ opinions on the appropriateness of the disciplinary response, for instance, “The degree of discipline administered to the child is appropriate.”

#### 3.2.4 Social evaluations of the teacher

Building upon established methodologies from prior studies (Wang and Murnighan, 2017; Zhang and Qi, 2024), we administered two validated items to measure participants’ perceptions of disciplinary teachers’ trustworthiness: “Wang has always been a steadfast support for the child.” and “Wang is highly reliable.” These items demonstrated strong internal consistency (*α* = 0.88). To evaluate interpersonal trust, we implemented three behavioral orientation measures, one of which stated: “I would like to share something about my child with Wang.” This scale yielded acceptable reliability (α = 0.78). Responses were collected using a standardized 5-point scale, with anchors ranging from 1 (strongly disagree) to 5 (strongly agree).

### 3.3 Data analysis

Statistical analyses were performed utilizing SPSS 24.0 software alongside the PROCESS macro package. The methodological framework comprised two sequential phases. First, preliminary analyses included conducting MANOVA on trustworthiness and parental trust. The second phase involved implementing Model 7 from the PROCESS toolkit to investigate moderated mediation effects, constructing a 95% bootstrap confidence interval through 5,000 iterative resampling procedures.

## 4 Results

### 4.1 Manipulation check

The operationalization of all variables in the current investigation was validated. An independent samples t-test examining violation severity demonstrated a significant main effect [*t*(460) = −3.67, *p* < 0.01, *d* = 0.34], with parental perceptions of high-severity violations (*M* = 4.30, *SD* = 0.81) substantially exceeding those of low-severity violations (*M* = 4.01, *SD* = 0.88). Subsequent between-subjects ANOVA for punishment intensity revealed a significant main effect [*F*(2,459) = 20.44, *p* < 0.01, η^2^ = 0.08]. Post-hoc comparisons indicated markedly higher intensity ratings for severe punishment (*M* = 3.32, *SD* = 0.10) relative to both mild (*M* = 2.62, *SD* = 0.10) and no punishment conditions (M = 2.43, SD = 0.11), *p*s < 0.01, though the latter two conditions did not differ significantly, *p* > 0.05. An ANOVA for disciplinary appropriateness similarly demonstrated significance [*F*(2,459) = 16.48, *p* < 0.01, η^2^ = 0.07], with appropriateness ratings for non-punitive responses (*M* = 3.49, *SD* = 0.10) being significantly inferior to both mild (*M* = 4.12, *SD* = 0.07) and severe punishment (*M* = 3.98, *SD* = 0.08), *p*s < 0.01, while no significant disparity emerged between the two punitive conditions, *p* > 0.05.

### 4.2 Preliminary analyses

**Figure 2:**
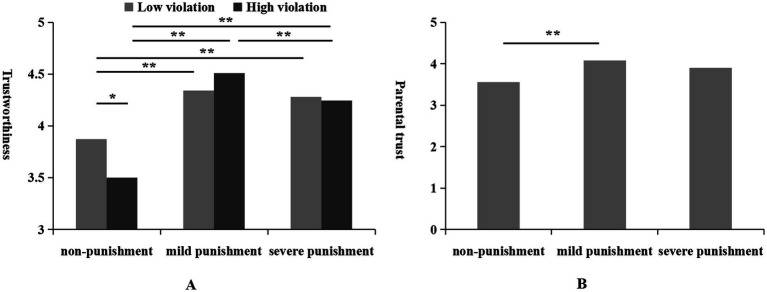
The effects of punishment on ratings of trustworthiness **(A)**, and parental trust **(B)**; The horizontal line represents the comparison between different conditions. *p < 0.05, **p < 0.01.

A 3 (punishment intensity) × 2 (violation severity) MANOVA on trustworthiness revealed that the main effects of punishment intensity was significant [*F*(2,456) = 27.17, *p* < 0.01, η^2^ = 0.11]. Teachers who administered no punishment received significantly lower trustworthiness scores (*M* = 3.69, SD = 0.08) compared to those who administered mild (*M* = 4.43, *SD* = 0.07) or severe punishment (*M* = 4.26, *SD* = 0.07), *p*s < 0.01. There was no significant difference in trustworthiness scores between teachers who administered mild and severe punishments, *p* > 0.05. The interaction between punishment intensity and violation severity was also significant [*F*(2,456) = 3.48, *p* < 0.05, η^2^ = 0.02]. Simple effects analysis revealed that under conditions of no punishment, the severity of the violation had a significant effect on trustworthiness, *F*(1,456) = 5.59, *p* < 0.05. Specifically, teachers who did not punish for low violations (*M* = 3.88, *SD* = 0.11) were rated higher in trustworthiness than those who did not punish for high violations (*M* = 3.50, *SD* = 0.11). No significant effect of violation severity was observed under conditions of mild or severe punishment, *F*s(1,456) < 1.26, *p*s > 0.05 (see Figure 2A). Simple effects analysis from the perspective of punishment intensity showed that in the context of low violations, the main effect of punishment intensity was significant [*F*(2,456) = 5.90, *p* < 0.01, η^2^ = 0.03]. Teachers who did not administer punishment (*M* = 3.88, *SD* = 0.10) received significantly lower trustworthiness scores compared to those who administered mild (*M* = 4.34, *SD* = 0.09) or severe punishment (*M* = 4.28, *SD* = 0.10), *p*s < 0.01, with no significant difference between the latter two, *p* > 0.05.

A 3 (punishment intensity) × 2 (violation severity) MANOVA on parental trust indicated that only the main effect of disciplinary intensity was significant [*F*(2,456) = 5.46, *p* < 0.01, η^2^ = 0.03]. Specifically, parents exhibited significantly lower trust in teachers who did not administer any punishment (*M* = 3.48, *SD* = 0.10) compared to those who administered mild punishment (*M* = 3.89, *SD* = 0.08), *p* < 0.01. No significant differences were observed in trust scores between the mild punishment and severe punishment conditions (*M* = 3.73, *SD* = 0.09) or between the no punishment and severe punishment conditions, *p*s > 0.05 (see Figure 2B). Additionally, no other main effects or interactions reached statistical significance.

### 4.3 Moderated mediation effect analysis

**Figure 3:**
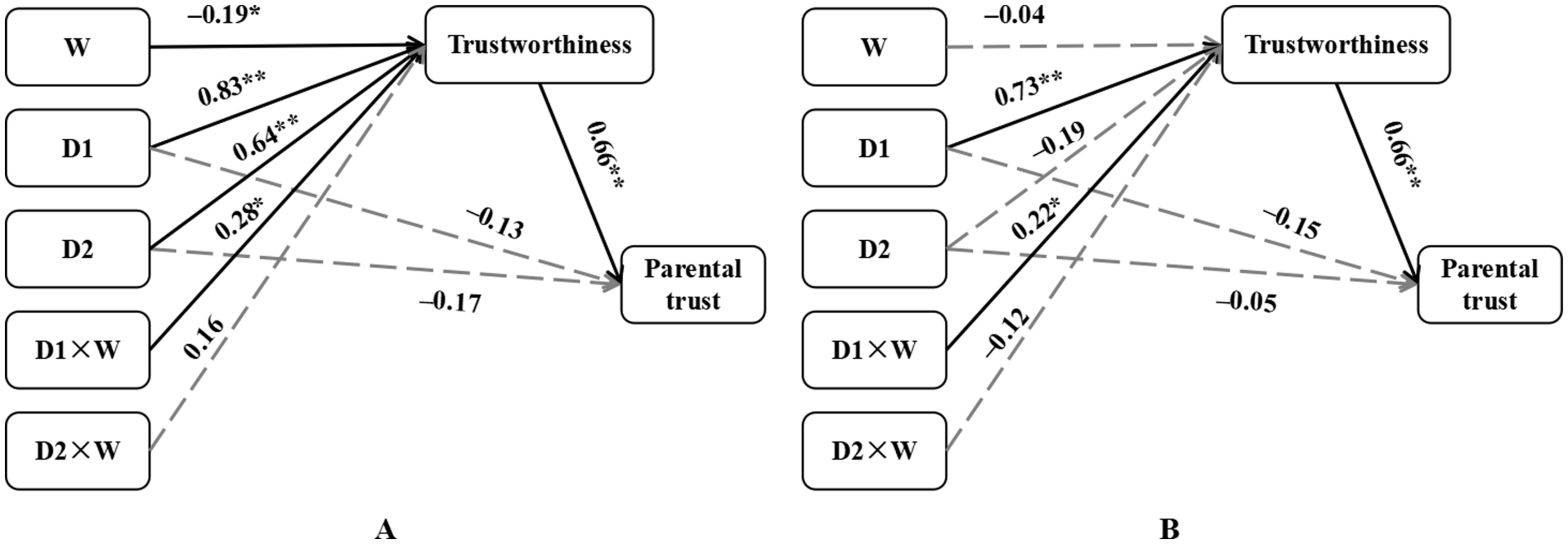
Process diagram for moderated mediation effect. **(A)** Testing whether trustworthiness mediates the effects of mild versus no punishment (D1) or severe versus no punishment (D2) on parental trust and the moderating of violation severity (W). **(B)** Testing whether trustworthiness mediates the effects of punishment versus no punishment (D1) or severe versus mild punishment (D2) on parental trust and the moderating of violation severity (W). All the regression coefficients are standardised, *p < 0.05, **p < 0.01.

The study incorporated parental characteristics (identity and age), student characteristics (gender, age and Only-child status), and family characteristics (Hukou classification and Family mobility) as control variables, disciplinary intensity as the independent variable with multiple classifications, parental trust as the dependent variable, trustworthiness as the mediating variable, and severity of violation as the moderating variable. To examine the mediating effect of trustworthiness on the relationship between disciplinary intensity and parental trust, as well as the moderating effect of violation severity, we utilized Hayes (2017) SPSS macro PROCESS Model 7. Initially, an Indicator coding model analysis compared mild or severe discipline against no discipline (see Table 2). The results indicated that mild punishment significantly positively predicted parents’ perceived trustworthiness of disciplinary teachers (β = 0.83, *t* = 7.31, *p* < 0.01), while severe punishment also significantly positively predicted parents’ perception of disciplinary teachers’ trustworthiness (β = 0.65, *t* = 5.59, *p* < 0.01). Furthermore, the severity of the violation negatively predicted parents’ trust in disciplinary teachers (β = −0.19, *t* = −2.13, *p* < 0.05). The interaction between mild punishment and violation severity significantly predicted parents’ trustworthiness of disciplinary teachers (β = 0.28, *t* = 2.47, *p* < 0.05). Trustworthiness emerged as a significant positive predictor of parental trust (β = 0.66, *t* = 17.35, *p* < 0.01). These findings suggest that the relationship between mild discipline and trustworthiness is moderated by the severity of the violation. Bootstrapped conditional effects at high/low levels was used to tested the conditional indirect effects. Further analysis revealed that the conditional mediation effect was significant under different levels of violation severity. Specifically, for perceived trustworthiness, the indirect effect of mild punishment under low violation severity conditions was 0.36, with a 95% confidence interval [0.15, 0.59], excluding zero; under high violation severity conditions, the indirect effect was 0.73, with a 95% confidence interval [0.50, 0.97], also excluding zero. In summary, when faced with students’ infractions such as being 10 min late or missing one class, mild discipline increased parental trust more than no discipline by enhancing trustworthiness, with this indirect effect being more pronounced for more severe violation. Figure 3A illustrates the path diagram of the model.

**Table 2:** Testing the moderated mediation model (*N* = 462).

Regression Equation		Indicator Coding ^a^				Helmert Coding ^b^			
Outcome variable	Prediction variable	*R*^2^	β	*t*	95%CI	*R*^2^	β	*t*	95%CI
Trustworthniess	D1	0.14	0.83	7.31**	[0.60 1.05]	0.14	0.73	7.26**	[0.53 0.93]
	D2		0.64	5.59**	[0.41 0.86]		−0.19	−1.83	[−0.40 0.01]
	Violation Severity		−0.19	−2.13*	[−0.36–0.01]		−0.04	−0.85	[−0.13 0.05]
	D1 × Violation Severity		0.28	2.47*	[0.06 0.50]		0.22	2.18*	[0.02 0.42]
	D2 × Violation Severity		0.16	1.41	[−0.06 0.39]		−0.12	−1.11	[−0.32 0.09]
Parental Trust	D1	0.43	−0.13	−1.31	[−0.32 0.06]	0.43	−0.15	−1.74	[−0.32 0.02]
	D2		−0.17	−1.82	[−0.36 0.01]		−0.05	−0.55	[−0.21 0.12]
	Trustworthiness		0.66	17.35**	[0.58 0.73]		0.66	17.35**	[0.58 0.73]

Dummy coding of multicategorical independent variables: ^a^D1 = no punishment (−0.66), mild punishment (0.33), and severe punishment (0.33); D2 = no punishment (0), mild punishment (−0.50), and severe punishment (0.50). ^b^D1 = no punishment (0), mild punishment (1), and severe punishment (0); D2 = no punishment (0), mild punishment (1), and severe punishment (1). *p < 0.05, **p < 0.01.

Second, Helmert coding was employed to implement the analytical model, comparing punitive interventions (mild and severe) with non-punitive approaches, and comparing mild discipline with severe discipline (see Table 2). The analysis demonstrated that D1 (punishment vs. no punishment) served as a significant predictor positively associated with parental perceptions of trustworthiness (β = 0.73, *t* = 7.26, *p* < 0.01). Notably, the D1 × violation severity interaction term significantly influenced parental trust in disciplinary condition (β = 0.22, *t* = 2.18, *p* < 0.05). The trustworthiness exhibited a robust positive correlation with parental trust (β = 0.66, *t* = 17.35, *p* < 0.01). These outcomes indicate that violation severity moderates the association between disciplinary actions (D1: punishment vs. non-punishment) and perceived trustworthiness. Subsequent examination identified significant conditional mediation effects across varying punishment intensities. Quantitatively, under minor infractions (e.g., 10-min tardiness), D1’s indirect effect measured 0.34 (95% CI [0.15, 0.55]); whereas for major violations (e.g., class absenteeism), the indirect effect intensified to 0.63 (95% CI [0.40, 0.87]). This pattern suggests that punitive actions enhance parental trust through trustworthiness more effectively than non-intervention, particularly in severe misconduct scenarios. The structural relationships are graphically represented in Figure 3B.

## 5 Discussion

Based on a situational approach, this study is the first to investigate the association between teacher discipline intensity and parental trust in rural China, along with its underlying cognitive mechanisms. The findings provide initial evidence of a nonlinear relationship between teacher discipline intensity and parents’ perceived trustworthiness and trust in teachers in rural China. Specifically, an inverted U-shaped relationship between discipline intensity and perceived trustworthiness becomes significant when students exhibit more serious disciplinary issues. Moreover, compared to no discipline, both mild and severe discipline can enhance parents’ trust by increasing their perception of teachers’ trustworthiness. Importantly, the mediating effect of perceived trustworthiness is more pronounced when students display more serious disciplinary behaviors.

For perceived trustworthiness and trust behavior, the current study found that both mild and severe punishment conditions resulted in significantly higher evaluation scores compared to the no-punishment condition. However, there was no significant difference between the mild and severe punishment conditions. This finding provides partial support for Hypothesis 1 and aligns with signaling theory, indicating that disciplinary actions can influence bystanders’ moral judgments of the punisher (Gintis et al., 2001). In line with previous research (Spadaro et al., 2023; Wang and Murnighan, 2017; Zhang and Qi, 2024), mild punishment led to higher trustworthiness scores and increased trusting behaviors compared to no punishment. This suggests that rural parents in China acknowledge and endorse the correction of students’ deviant behavior through appropriate disciplinary actions, perceiving teachers who implement such actions as more reliable and trustworthy (Qin et al., 2022; Wang et al., 2021). An intriguing finding was that parents exhibited comparable levels of trustworthiness and trust behaviors toward teachers irrespective of the severity of the discipline administered. This phenomenon can be attributed to rural Chinese parents’ ability to discern the intensity differences between mild and severe punishments while perceiving both methods as equally appropriate, as supported by the analysis of punishment appropriateness. Furthermore, this observation highlights the deep-seated respect, acceptance, and tolerance of teachers’ disciplinary authority among rural Chinese parents (Wang et al., 2021).

Current research has demonstrated that disciplinary intensity significantly and positively predicts perceived trustworthiness, which in turn positively predicts parental trust. A mediation analysis of categorical variables revealed that compared to no punishment, mild, severe, and overall punishment can enhance parental trust by improving perceived trustworthiness. Conversely, the mediating effect between severe and minor punishment is not statistically significant. These findings partially support the study’s hypothesis 2 and corroborate the principles of signaling theory (Gintis et al., 2001), suggesting that punishment can convey the moral character of the punisher, influence their reputation, and ultimately alter bystanders’ trust behaviors. Consistent with prior studies using economic game tasks (Spadaro et al., 2023), this indicates that discipline can significantly predict trustworthiness within an appropriate scope. Additionally, these results align with previous organizational management research, demonstrating that trustworthiness mediates the relationship between managerial punishment and bystander trust (Wang and Murnighan, 2017; Zhang and Qi, 2024). Thus, the perceived trustworthiness of educators may function as an intermediary variable that explains the association between pedagogical discipline strategies and parental trust in teachers. In addition, despite the relatively large path coefficient of D2 (mild vs. severe punishment) for trustworthiness under the Helmert coding framework, it does not achieve statistical significance. This might suggest that parents may prioritize the concept of punishment itself over its intensity. However, in our opinion, this phenomenon may arise from the specific nature of severe punishment for misconduct in the research context. Within the educational framework of China, summoning parents is considered a stringent disciplinary measure and is typically employed only when student infractions are relatively grave. Nevertheless, from the standpoint of parents, being summoned to school can also be viewed as a form of communication or collaboration between the home and the school. At the very least, teachers inform parents about students’ violations and consult with them on addressing such issues. Consequently, this potential positive aspect may offset the adverse effects caused by excessive punishment, thereby contributing to the aforementioned inconclusive results.

The current experiment also revealed the moderating effect of the severity of student violations on the relationship between disciplinary intensity and parents’ perceived trustworthiness, thereby supporting Hypothesis 3. Specifically, both mild and overall punishments significantly increased parents’ perceived trustworthiness compared to no punishment, irrespective of the degree of student misconduct. However, the mediating effect of perceived trustworthiness was more pronounced when students committed more severe violations. This finding aligns with key principles of signaling theory (Gintis et al., 2001) and the deserved theory of justice (Mooijman and Graham, 2018), indicating that proportionate punishment can effectively communicate the moral integrity of the authority figure, thereby enhancing bystanders’ interpersonal trust. Consistent with our findings, Wang and Murnighan (2017) demonstrated in an enterprise management context that mild punishment significantly improves bystanders’ perceived trustworthiness compared to no punishment, with a more substantial mediating effect observed for more serious employee misconduct. Therefore, teachers’ punishment of severely transgressive children, as opposed to minor infractions, can significantly predict perceived trustworthiness and consequently bolster parents’ trust in educators.

## 6 The practical implications

To our knowledge, this study uses the experimental method for the first time to examine the spillover effects of teacher disciplinary practices on parental trust within educational frameworks. The findings carry substantial theoretical and practical relevance for advancing sustainable family-school partnerships in contemporary China. In a theoretical sense, this investigation introduces methodological innovation by establishing causal relationship between pedagogical discipline and parental trust through controlled experimentation, while delineating the cognitive mechanisms and contextual thresholds governing the impact of disciplinary intensity. These contributions meaningfully extend the theoretical domains of signaling theory and organizational justice frameworks. Furthermore, empirical evidence demonstrates that parents systematically associate disciplinary interventions with enhanced teacher trustworthiness, particularly regarding severe behavioral infractions, reflecting rural Chinese communities’ endorsement of pedagogically justified disciplinary actions.

These insights provide an operational framework for addressing the prevalent professional dilemma characterized by educators’ reluctance, hesitancy, and perceived incapacity in the management of students’ disciplinary behavior. Specifically, when implementing disciplinary actions, teachers can enhance the relationship between home and school by focusing on the following aspects. First, to ensure the legitimacy, appropriateness, and fairness of disciplinary measures, a comprehensive evaluation should be conducted based on the specific circumstances of the student who has violated the rules (such as the nature of the violation, underlying motives, and frequency of infractions). Subsequently, disciplinary actions that are acceptable to teachers, parents, and students should be implemented. Second, it is crucial to remember that the primary purpose of punishment is to facilitate the holistic development of children rather than to inflict suffering or harm. Therefore, when administering discipline, attention must also be given to humanistic care and educational outcomes. Only through such an approach can the ultimate goal of educational discipline be effectively achieved.

## 7 Limitations and future recommendations

The present study, like prior research, is subject to several methodological constraints. First, the present research findings are solely based on the parent population of a junior high school in a rural area. The sampling lacks representativeness, thereby not only constraining the generalizability and universality of the results but also precluding the possibility of comparing differences between urban and rural populations. Consequently, future studies could consider utilizing publicly available databases with nationally representative samples (Zhang et al., 2025b) or datasets encompassing diverse educational stages (e.g., higher education or preschool education) to further validate the robustness of the findings. Second, the current experiment asks parents to make self-reported reports based on hypothetical disciplinary events, and their responses may be influenced by social desirability and cognitive bias. Therefore, future research could use real cases and incorporate forms such as other-assessment reports to enhance the authenticity and objectivity of the research data. Third, the current study involves only a group of Chinese parents, and the sample is relatively homogenous. However, there are significant cultural differences in the philosophy and development of educational discipline. A study conducted in the United States suggests that empathic discipline can enhance home-school communication and build parental trust (Okonofua et al., 2016). Consequently, future research could embark on a cross-cultural, large-sample survey study.

## 8 Conclusion

The present study investigates the cognitive mechanisms through which disciplinary intensity influences interpersonal trust among parents in rural China. The findings demonstrate that teacher discipline not only directly strengthens parental trust but also indirectly increases it through perceived trustworthiness. Notably, the severity of student misconduct moderates the relationship between disciplinary intensity and parental trust, indicating that rigorous disciplinary behaviors in response to severe violations foster heightened perceived trustworthiness relative to less stringent interventions.

## Data Availability

The raw data supporting the conclusions of this article will be made available by the authors, without undue reservation.
